# A Randomized Controlled Trial Comparing High- and Moderate-Intensity Interval Walking on Hematological and Functional Markers in Postmenopausal Women with Obesity

**DOI:** 10.3390/sports14040149

**Published:** 2026-04-13

**Authors:** Wissal Abassi, Nejmeddine Ouerghi, Georges Jabbour, Moncef Feki, Anissa Bouassida, Mykolas Deikus, Jolita Vveinhardt, Antonella Muscella

**Affiliations:** 1 Research Unit “Sport Sciences, Health and Movement” (UR22JS01) High Institute of Sport and Physical Education of Kef, University of Jendouba, Kef 7100, Tunisia; wissalabassi93@gmail.com (W.A.); najm_ouerghi@hotmail.com (N.O.); bouassida_anissa@yahoo.fr (A.B.); 2 Faculty of Medicine of Tunis, University of Tunis El Manar, Rabta Hospital, LR99ES11, Tunis 1007, Tunisia; monssef.feki@gmail.com; 3 Sport Coaching Department, College of Sport Sciences, Qatar University, Doha 2713, Qatar; gjabbour@qu.edu.qa; 4 Research Center on Marriage and Family, Vytautas Magnus University, 44260 Kaunas, Lithuania; mykolas.deikus@vdu.lt; 5 Klaipėdos Valstybinė Kolegija/Higher Education Institution, 91274 Klaipeda, Lithuania; j.vveinhardt@kvk.lt; 6 Department of Biological and Environmental Science and Technologies (DiSTeBA), University of Salento, 73100 Lecce, Italy

**Keywords:** postmenopausal women, hematological markers, muscle damage, body composition, 6 min walk test

## Abstract

Postmenopausal women with obesity often show blood abnormalities and low plasma volume, which reduce aerobic capacity and raise health risks. The purpose is to compare the effects of high-intensity (HIIWT) versus moderate-intensity interval walking training (MIIWT) on body composition, plasma volume variations (PVV), hematological parameters, muscle damage, and aerobic capacity in postmenopausal women with overweight/obesity. Thirty-two postmenopausal women with overweight/obesity were randomly assigned to HIIWT (*n* = 11), MIIWT (*n* = 11), or control (CON, *n* = 10) groups. The HIIWT and MIIWT groups performed intermittent walking at 90–110% and 60–80% of their 6-min-walk-test (6MWT) distance, respectively, four times per week for 10 weeks. Body composition, hematological and muscle damage markers, and 6MWT performance were assessed pre- and post-intervention. After ten weeks, PVV was calculated in all three groups. A significant group × time interaction was observed for body composition, erythrocytes, hemoglobin levels, hematocrit, creatine kinase (CK), lactate dehydrogenase (LDH), and 6MWT performance (*p* < 0.05). Both the HIIWT and MIIWT groups showed significant reductions in body mass, body fat, waist circumference (*p* < 0.05), and erythrocyte count (*p* = 0.010 and 0.028, respectively). Only the HIIWT group showed significant reductions in hemoglobin (*p* < 0.001), hematocrit (*p* = 0.005), CK (*p* = 0.002), and LDH (*p* = 0.009), along with a significant increase in 6MWT-performance (*p* = 0.002). The HIIWT group demonstrated a significantly greater increase in PVV compared to both MIIWT (*p* = 0.018) and CON (*p* < 0.001) groups. HIIWT induced superior improvements in body composition, aerobic capacity, plasma volume, and hematological and muscle-damage markers compared to MIIWT. HIIWT represents a practical strategy for improving health outcomes in postmenopausal women with overweight/obesity.

## 1. Introduction

Aerobic performance constitutes a fundamental component of overall health, as it is closely associated with enhanced cardiovascular function, improved metabolic regulation, and a reduced risk of chronic disease [1]. The physiological basis of aerobic capacity includes cardiac output, blood volume, and hematological parameters such as hemoglobin concentration, hematocrit, and red blood cell count [2,3]. In particular, plasma volume expansion reduces blood viscosity through hemodilution, thus facilitating oxygen transport and improving cardiovascular efficiency [4,5]. In contrast, a reduction in plasma volume increases blood viscosity, impairs circulatory flow, and increases cardiovascular effort, factors that could increase the risk of hemodynamic instability, especially in conditions of physical or clinical stress [6,7,8,9].

The co-occurrence of obesity and menopause-related hormonal changes contributes to marked variability in plasma volume [8,10,11]. Declining estrogen levels during menopause affect vascular function, renal sodium balance, red blood cell count, hemoglobin concentration, fluid distribution, hematocrit, and plasma viscosity [12]. In individuals with obesity, high body mass is inversely correlated with plasma volume, as observed in adolescent boys [10,13], and adult women with obesity show significant reductions in plasma volume, with menopausal status being an independent predictor [14]. In post-menopausal women with obesity, reduced plasma volume can impair oxygen delivery, increase cardiovascular strain, and limit exercise tolerance, making it a critical factor for overall cardiovascular and metabolic health [7,8].

Exercise interventions such as high-intensity intermittent walking (HIIT) or moderate-intensity intermittent walking (MIIWT) could influence plasma volume by inducing hemodynamic and hormonal adaptations. Repeated exposure to elevated cardiac output and mechanical stress during these protocols may promote plasma volume expansion and improve fluid distribution, as observed in studies reporting acute and chronic increases in plasma volume after high-intensity interval exercise [13,15]. High-intensity interval training has been shown to increase plasma volume responses and attenuate age-related declines in PV, supporting potential benefits in cardiovascular function and oxygen delivery [14].

These adaptations could mitigate the cardiovascular and metabolic limitations associated with reduced plasma volume in postmenopausal women with obesity, although the specific effects in this population remain largely unexplored, whereas plasma volume expansion is a well-established training adaptation in healthy young adults [8,15]. Differences in exercise intensity are anticipated to elicit distinct hemodynamic, hormonal, and metabolic responses, which may differentially influence plasma volume. HIIT imposes greater cardiovascular stress, higher cardiac output, and increased shear forces compared with moderate-intensity exercise, leading to more pronounced PV expansion and fluid redistribution over time [16,17,18]. These intensity-dependent responses are mediated by greater stimulation of the autonomic nervous system, enhanced release of vasodilatory metabolites, and more substantial intravascular fluid shifts, potentially resulting in more robust adaptations in plasma volume and cardiovascular function.

High-intensity interval training has emerged as a promising, time-efficient strategy for improving health outcomes, particularly cardiometabolic health and body composition in populations at increased metabolic risk. In adolescents with overweight or obesity, HIIT has been shown to improve cardiometabolic biomarkers and physical fitness parameters [19,20]. Similarly, in postmenopausal women with metabolic syndrome, HIIT favorably influences metabolic and cardiovascular risk markers [21]. Although the strongest evidence concerns overweight and clinical populations, meta-analytic data indicate that HIIT can also produce improvements in body fat, aerobic capacity (VO_2_max), and cardiovascular risk factors in broader adult samples, including normal-weight individuals [22].

This training modality has been associated with positive changes in body composition, reductions in systemic inflammation, and improvements in cardiometabolic risk [20,21,23]. Furthermore, HIIT may also contribute to plasma volume expansion in postmenopausal women with obesity, suggesting its potential role in improving hemodynamic regulation within this population [8]. Despite its benefits, HIIT’s demanding nature may limit adherence, particularly in populations with reduced fitness or motivation; in previous research, moderate intensity interval training has been identified as a promising alternative to HIIT, offering greater adherence and comparable health benefits [24]. Specifically, Coquart et al. [25] reported that postmenopausal women with obesity were more inclined to engage in moderate intensity interval walking training, primarily due to its greater variability and lower monotony compared to continuous training. In the present study, adherence was high in both groups (97% of scheduled sessions completed), suggesting that, despite its higher intensity, HIIWT was feasible and well-tolerated in this population. These findings indicate that, when appropriately supervised and individualized, even high-intensity protocols can achieve adherence comparable to moderate-intensity training

High-intensity exercise may also induce acute skeletal muscle stress, reflected by transient increases in circulating muscle damage markers such as creatine kinase (CK) and lactate dehydrogenase (LDH) [26,27,28]. CK and LDH responses vary with exercise intensity, duration, mode, and individual training status, with higher intensities generally producing larger transient elevations post-exercise. In sedentary and overweight populations, monitoring these markers is particularly relevant to evaluate exercise safety, muscular adaptation, and the balance between physiological remodeling and excessive tissue stress [29,30,31]. However, the chronic effects of different interval walking intensities on muscle damage markers in postmenopausal women with obesity, remain poorly understood [32,33].

However, no studies to date have directly compared the effects of different interval walking training modalities on hemodynamic adaptations, particularly plasma volume regulation in postmenopausal women with obesity. This represents a significant gap in the literature, especially considering the physiological and clinical relevance of plasma volume changes in this population. Our study aims to address this gap by evaluating and comparing the effects of high-intensity interval walking training (HIIWT) and moderate-intensity interval walking training (MIIWT) interventions on body composition, hematological parameters, plasma volume variations, muscle damage markers, and aerobic capacity, in postmenopausal women with overweight/obesity. We hypothesize that both HIIWT and MIIWT will lead to improvements in body composition, hematological markers, and aerobic capacity in postmenopausal women with overweight/obesity. However, we expect that HIIWT will induce greater plasma volume expansion and more pronounced hemodynamic and aerobic adaptations compared to MIIWT, whereas MIIWT will demonstrate higher adherence and lower markers of muscle damage.

## 2. Materials and Methods

### 2.1. Sample Size Calculation

The required sample size was determined using G*Power 3.1 software, based on parameters derived from a previous study [34]. Assuming a partial eta-squared effect size of 0.38, a statistical power (1-β) of 0.90, and an alpha level of 0.05, the minimum sample size was estimated to be 27 participants. Ultimately, 32 participants were enrolled in the study.

### 2.2. Participants

In total, 47 women were initially screened for eligibility based on the following criteria: (1) age range of 45–59 years; (2) postmenopausal women (absence of menses > 12 months); (3) body mass index (BMI) ≥ 25 kg/m^2^; (4) body weight remained constant (±2 kg) during the past 3 months; (5) stable eating habits and physical activity for at least 3 months; (6) sedentary lifestyle (exercise less than 1 h/week). Exclusion criteria included medical contraindications to physical activity, diagnosed metabolic, hormonal, orthopedic, or cardiovascular conditions, and current use of hormone replacement therapy. Additionally, participants using medications known to directly affect hematological parameters or plasma volume—such as diuretics—were excluded. Common medications for hypertension, dyslipidemia, or other conditions that do not influence the study outcomes were allowed, and anticoagulants were excluded if there were safety concerns related to exercise.

**Figure 1:**
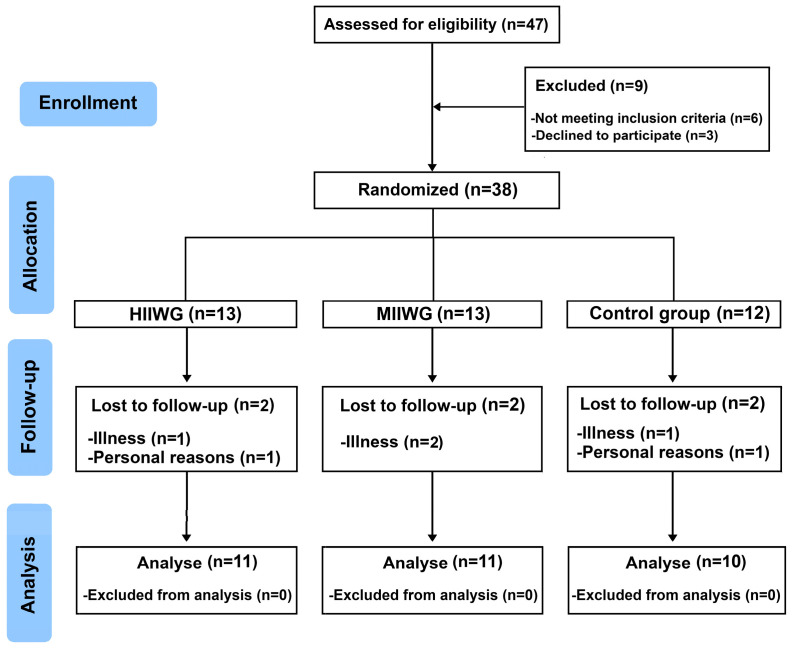
Flow-chart of study. HIIWG, high-intensity interval walking group; MIIWG, moderate-intensity interval walking group.

Of the 47 women screened, six did not meet the inclusion criteria, and three declined to participate due to personal reasons. The remaining 38 participants were randomly allocated to three groups: (1) high-intensity intermittent walking training group (HIIWG, *n* = 13), (2) moderate-intensity intermittent walking training group (MIIWG, *n* = 13), and (3) control group (CON, *n* = 12). During the intervention period, six participants withdrew, two from each group, for reasons not related to the study protocol. By the end of the study, a total of 32 participants had completed the intervention, with 11 individuals in each experimental group and 10 in the CON (HIIWG, *n* = 11; MIIWG, *n* = 11; CON, *n* = 10) (Figure 1). Baseline characteristics were similar across groups, confirming homogeneity before the intervention.

All outcome analyses, including PVV, hematological parameters, and functional performance, were conducted on participants who completed the full 10-week intervention (completer-only analysis; HIIWG, *n* = 11; MIIWG, *n* = 11; CON, *n* = 10). Participants who withdrew during the study were excluded from post-intervention analyses because incomplete data did not allow accurate calculation of outcomes. Adherence among completers was high (97% of scheduled sessions completed), and withdrawals were unrelated to the intervention, minimizing potential bias. This approach ensures that the analysis reflects reliable and robust intervention effects.

All participants provided written informed consent after receiving detailed information regarding the study’s purpose, potential benefits, and associated risks. The study was conducted by the guidelines contained in the Declaration of Helsinki and received approval from the Scientific and Ethics Committee of the High Institute of Sports and Physical Education of Kef (ISSEPK) ISSEPK-0031/2024. The trial was retrospectively registered on ClinicalTrials.gov (NCT07019285) due to administrative timing between ethics approval and registry submission; all outcomes and timepoints were pre-defined in the approved protocol, and no post hoc changes were made. 

ClinicalTrials.gov Identifier: NCT07019285, 12 June 2025 (retrospectively registered).

### 2.3. Procedures

This randomized controlled trial was conducted over a 10-week period between January and March 2025. Assessments were performed before (baseline) and after the intervention.

The assessments of body composition, hematological parameters [erythrocytes, hemoglobin (Hb) and hematocrit (Ht), mean corpuscular volume (MCV), mean corpuscular hemoglobin concentration (MCHC), and mean hemoglobin content (MHC)], muscle damage markers [creatine kinase (CK) and lactate dehydrogenase (LDH)] and aerobic capacity [6 min walk test (6MWT)] took place one week before (baseline) and after (post) completion of the intervention and were considered secondary outcomes. Plasma volume variations (PVV), defined as the primary outcome, were calculated after ten weeks under the three conditions (HIIWT, MIIWT and CON).

After baseline assessments, participants were randomly assigned to one of three groups: HIIWG; MIIWG, or CON. Randomization was conducted using a computer-generated list created with Microsoft Excel, ensuring a 1:1:1 allocation ratio. The randomization sequence was generated by an independent researcher who was not involved in the enrollment, assessment, or training of participants. Group assignments were concealed in sealed, opaque, and sequentially numbered envelopes to maintain allocation concealment. Envelopes were opened only after baseline measurements were completed. Due to the nature of the intervention, participants and trainers could not be blinded to group allocation. However, outcome assessors and data analysts were blinded to group assignment to minimize bias. Baseline comparability between groups was verified using one-way analysis of variance (ANOVA), showing no significant differences across groups for the variables assessed (*p* > 0.05).

Attendance at all supervised training sessions was recorded, and participants received weekly reminders to enhance adherence. Outside the intervention, participants were instructed to maintain their usual physical activity and diet. Physical activity levels were self-reported weekly using a questionnaire.

No specific dietary restrictions or interventions were applied, and nutritional status was monitored by asking participants to report any significant changes in their eating habits during the study period. Dietary intake was assessed using 3-day food diaries collected at baseline, mid-intervention (week 5), and post-intervention (week 10) and analyzed for energy intake and macronutrient composition using Nutritionist Pro™ nutrition analysis software version 7.9 (Axxya Systems, Fort Collins, CO, USA)

To control hydration, participants were instructed to maintain habitual fluid intake throughout the study and to consume approximately 500 mL of water 2 h prior to all laboratory assessments.

Adverse events were defined as any unintended physical effects or injuries related to the intervention. These were monitored systematically throughout the intervention period through direct observation by the supervising instructors and verbal questioning of participants at each session. No adverse events were reported during the study.

### 2.4. Body Composition Measures

Anthropometric measurements were conducted under standardized pre-test conditions. Participants fasted for at least 8 h, maintained usual hydration, and avoided strenuous exercise in the 24 h prior to testing. Measurements were performed at a consistent time of day to minimize diurnal variation. Anthropometric measurements were conducted according to standardized protocols, with participants wearing light clothing and no footwear. Body weight (kg) and body fat percentage were recorded using an electronic scale (Tanita BC-533, Tokyo, Japan). Height (m) was measured using a stadiometer (Holtain Ltd., Crymych, UK), and body mass index (BMI) was calculated as weight in kilograms divided by the square of height in meters (kg/m^2^). Waist circumference (cm) was measured to the nearest 0.1 cm using a non-elastic measuring tape, placed horizontally at the midpoint between the inferior margin of the last palpable rib and the superior border of the iliac crest, with participants standing upright and breathing normally.

### 2.5. Blood Sampling and Analysis

After an overnight fast, venous blood samples were collected at approximately 8:00 a.m. from the antecubital vein by a trained technician. Participants were instructed to avoid any vigorous physical activity for 48 h prior to each blood collection to minimize the acute influence of exercise on muscle damage markers (CK and LDH).

To assess hematological parameters, venous blood samples (5 mL) were collected into EDTA-containing vacutainer tubes. Hematological indices were analyzed using a multichannel automated hematology analyzer (XN450; Sysmex, Norderstedt, Germany). The primary outcome of the study was plasma volume variation (PVV), specified in the trial registry. Plasma volume variations (PVV) were calculated and expressed as percentage changes in plasma volume, based on Ht and Hb values, using the Costill and Fink (1974) method.

%PVV=100×[(Hb0/Hb1)×(100−Ht1)/(100−Ht0)]−1

where Hb0 and Ht0 are the pre-intervention values, and Hb1 and Ht1 are the post-intervention values. PVV represents an estimate of plasma volume changes and assumes stable red blood cell mass and hydration between measurements. This method provides a practical approach for assessing plasma volume responses in clinical populations but may be influenced by variations in hydration or iron status.

To assess biochemical indicators of muscle damage, blood samples were collected into lithium heparin tubes, centrifuged at 2000× *g* for 25 min, and subsequently analyzed using a Beckman Coulter AU480 Chemistry Analyzer (Beckman Coulter, Inc., Brea, CA, USA).

To ensure data accuracy, post-intervention concentrations of CK and LDH were adjusted for plasma volume variations based on these calculated values.

### 2.6. Exercise Training Protocols

For ten weeks, participants in the HIIWT and MIIWT groups trained four times per week, completing a total of 40 sessions. Each session included a standardized 15 min warm-up at 50% of the individual 6 min walk test distance (6MWTD) recorded at baseline, followed by 5 min of dynamic stretching, and concluded with a 10 min cool-down for relaxation and recovery. The total duration of each session was approximately 90 min

**Figure 2:**
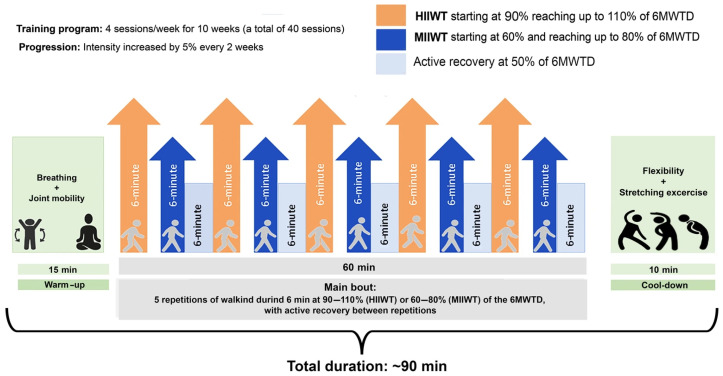
Schematic representation of the moderate- (MIIWT) and high-intensity (HIIWT) interval walking training protocols. Training intensities were based on the distance achieved during the 6 min walk test (6MWTD). Progression was applied every two weeks from week 3 through week 10. HIIWT, High-Intensity Interval Walking Training; MIIWT, Moderate-Intensity Interval Walking Training; 6MWTD, 6-Minute Walk Test Distance.

HIIWT participants performed five 6 min walking intervals at 90–110% of their baseline 6MWTD, with 6 min of active recovery between intervals. MIIWT participants completed five 6 min intervals at 60–80% of their baseline 6MWTD, also interspersed with 6 min of active recovery, following Guessogo et al. [35]. Training intensity was gradually increased: during the first two weeks, HIIWT and MIIWT participants trained at 90% and 60% of their 6MWTD, respectively, with increments of 5% of the baseline 6MWTD every two weeks from weeks three to ten (Figure 2). Exercise intensity was monitored using individual 6MWTD performance, with adherence to prescribed intensity confirmed by heart rate measurements and participants’ ratings of perceived exertion (RPE) recorded during each session. Overall, participants completed 97% of scheduled sessions, with no adverse events, indicating high compliance and tolerability.

### 2.7. Aerobic Capacity Measurements

The 6 min walk test (6MWT) was administered to assess exercise capacity, in accordance with the guidelines established by the American Thoracic Society (American Thoracic Society, 2002). Participants were familiarized with the test procedures prior to the actual assessment. The walking course was 30 m in length, marked at 5 m intervals with a tape on the floor, and was bounded at each end by two traffic cones. Participants were instructed to walk as far as possible at a self-paced speed for 6-min. Results were expressed as the total distance covered in meters. Each participant was tested individually and received standardized verbal encouragement throughout the test.

### 2.8. Statistical Procedures

Statistical analyses were performed using SPSS software, version 27.0 for Windows (SPSS, Chicago, IL, USA). Normality and homogeneity of variance were assessed using the Shapiro–Wilk and Levene’s tests, respectively, and both assumptions were confirmed. Baseline differences between groups were assessed using one-way analysis of variance (ANOVA). To assess the effects of the intervention, a two-way repeated measures ANOVA (3 groups × 2-time points: pre- and post-intervention) was performed to determine group × time interactions in body composition, hematological parameters and muscle damage markers across the HIIWG, MIIWG, and CON groups. Post hoc analyses using the Bonferroni test were conducted when a significant interaction was detected. Although multiple outcomes were assessed, plasma volume variation (PVV) was pre-specified as the primary outcome, and analyses related to PVV were considered confirmatory. All other outcomes, including body composition, hematological indices, muscle damage markers (CK, LDH), and functional performance (6MWT), were treated as secondary and exploratory endpoints. To limit Type I error, emphasis was placed on PVV, and effect sizes were interpreted alongside *p*-values to evaluate the robustness and practical relevance of the findings. Effect sizes (Cohen’s d) for body composition, hematological markers, and muscle damage indices refer to within-group pre–post changes, while effect sizes for PVV represent between-group differences at post-intervention. Effect sizes (ES) were interpreted as follows: 0.00 < d < 0.49 = small effect; 0.50 < d < 0.79 = moderate effect; d > 0.80 = large effect. Data were expressed as the mean ± standard deviation (SD). A value of *p* <0.05 was accepted as the minimal level of statistical significance.

## 3. Results

### 3.1. Dietary Monitoring

No specific dietary restrictions or interventions were applied during the study. Nutritional status was monitored by asking participants to report any significant changes in their eating habits. Dietary intake was assessed using 3-day food diaries collected at baseline, mid-intervention (week 5), and post-intervention, and analyzed for total energy intake and macronutrient composition (protein, fat, carbohydrates). No significant changes were observed over time or between groups, indicating that participants maintained their habitual dietary intake throughout the study (Table 1).

### 3.2. Body Composition, Aerobic Capacity, and Hematological Outcomes

Pre- and post-intervention values for body composition, aerobic capacity, and hematological markers across all three groups, and the results of the repeated measures ANOVA are presented in Table 2. No significant baseline differences were observed between the groups for any of the analyzed variables. No significant differences were detected in the CG between baseline and post-intervention measurements. All participants reported no substantial changes in their dietary habits during the study period, indicating that the observed changes in body composition and biochemical parameters were likely attributable to the exercise intervention.

### 3.3. Exercise Intensity Compliance

Training intensity was monitored via heart rate (HR) and ratings of perceived exertion (RPE) during each session. Participants in the HIIWT group increased from 65 ± 5% of HRmax at baseline to 87 ± 4% at post-intervention (*p* < 0.001), with RPE increasing from 10 ± 1 to 16 ± 1 (*p* < 0.001). Participants in the MIIWT group increased from 60 ± 4% HRmax to 68 ± 3% HRmax (*p* < 0.01), with RPE increasing from 9 ± 1 to 12 ± 1 (*p* < 0.01).

Adherence was high, with 97% of scheduled sessions completed and no adverse events reported, confirming that the intended high- and moderate-intensity targets were consistently achieved.

### 3.4. Body Composition and Aerobic Capacity

Following the intervention, both the HIIWG and MIIWG showed significant reductions in body mass (*p* < 0.001, d = 0.60 and *p* = 0.015, d = 0.13, respectively), BMI (*p* < 0.001, d = 0.50 and *p* = 0.014, d = 0.11, respectively), body fat percentage (*p* < 0.001, d = 0.26 and *p* = 0.016, d = 0.08, respectively), and waist circumference (*p* = 0.001, d = 0.71 and *p* = 0.011, d = 0.27, respectively). Lean mass remained stable in both groups (*p* > 0.05), indicating preservation of muscle mass.

However, a significant improvement in 6MWTD was observed after intervention, only in the HIIWG (*p* = 0.002, d = 0.36) (Table 2).

### 3.5. Hematological Markers and Plasma Volume

**Figure 3:**
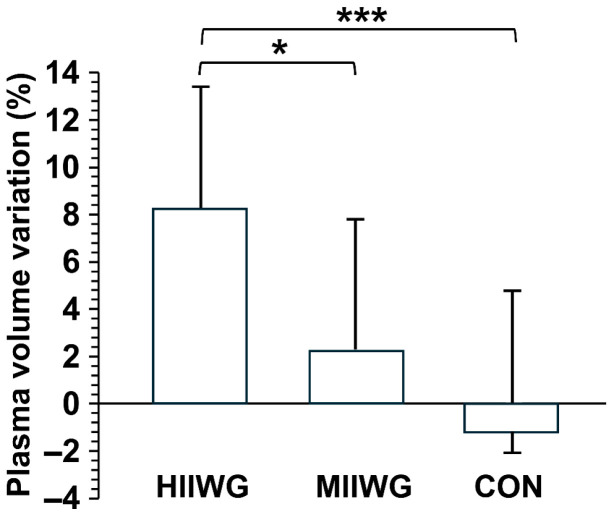
Plasma volume variation after the intervention in the high-intensity interval walking group (HIIWG) moderate-intensity interval walking group (MIIWG), and control group (CON). * *p* < 0.05; *** *p* < 0.001.

A significant interaction effect between group and time was found for erythrocytes, hemoglobin, and hematocrit levels, indicating differential changes across groups over the intervention period. Precisely, in within-group analyses, the HIIWG showed significant reductions in erythrocyte count (*p* = 0.010, d = 1.16), hemoglobin concentration (*p* < 0.001, d = 0.91) and hematocrit levels (*p* = 0.005, d = 0.58), whereas the MIIWG showed a significant decrease only in erythrocytes count (*p* = 0.028, d = 0.43). One-way ANOVA revealed a significant difference in plasma volume variation (PVV) among the three groups (F_(2,29)_ = 7.190, *p* = 0.003). The HIIWG showed a significantly greater increase in PVV compared to both the MIIWG (8.18 ± 5.69 vs. 1.98 ± 5.80%, *p* = 0.018, d = 1.14) and control group (8.18 ± 5.69 vs. −1.17 ± 5.87%, *p* < 0.001, d = 1.72). Notably, individual responses varied considerably within each group, with PVV changes in the HIIWG ranging from approximately 2.5% to 13.9%, and similar variability was observed for erythrocytes, hemoglobin, and hematocrit, highlighting the heterogeneity of physiological adaptations among participants (Figure 3). Despite erythrocyte count, hemoglobin concentration and hematocrit levels reductions, the post-intervention absolute values remained within the clinically normal range for women.

### 3.6. Muscle-Damage Markers

**Figure 4:**
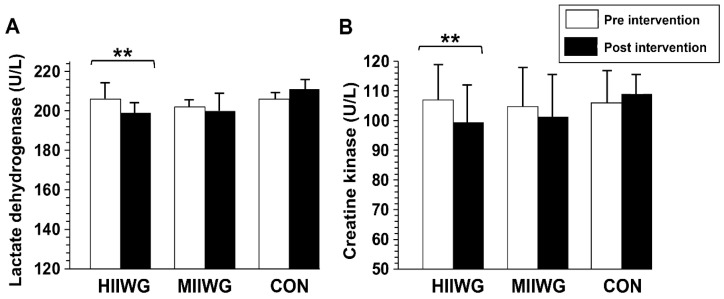
Lactate dehydrogenase (**A**) and creatine kinase (**B**) measured before and after the intervention in the high-intensity interval walking group (HIIWG) moderate-intensity interval walking group (MIIWG), and control group (CON). ** *p*  <  0.01; significant pre–post differences observed in the HIIWG.

A significant group × time interaction was observed for both CK and LDH levels (*p* < 0.05). Post hoc analysis revealed a significant reduction in CK (*p* = 0.002, d = 0.51) and LDH levels (*p* = 0.009, d = 0.40) in the HIIWG compared to the other groups (Figure 4).

## 4. Discussion

The purpose of this study was to compare the effects of high-intensity interval walking training (HIIWT) and moderate-intensity interval walking training (MIIWT) on various health markers in postmenopausal women with overweight or obesity. The main findings showed that although both training protocols effectively improved body composition, only HIIWT improved hematological markers, plasma volume variations (PVV), muscle damage indicators, and aerobic capacity compared to MIIWT, confirming our hypothesis that HIIWT would induce greater hemodynamic and aerobic adaptations than MIIWT. To our knowledge, this is the first study to evaluate the effect of interval walking training at different intensities on hematological markers in postmenopausal women with obesity. Our findings revealed that the HIIWT program resulted in a significant decrease in erythrocytes count, hematocrit, and hemoglobin concentration, accompanied by a marked increase in plasma volume. In contrast, the MIIWT intervention led to a reduction only in erythrocyte count. Importantly, post-intervention values remained within the clinically normal range for women, indicating a favorable hemodilution response rather than pathological anemia.

To date, only a limited number of studies have assessed PVV in women with obesity, especially in postmenopausal individuals, despite their heightened cardiovascular risk [8]. Our HIIWT protocol (90–110% of individual 6MWT velocity) does not fully match traditional HIIT definitions (≥85% HRR or ≥90% VO_2_max), particularly those typically used in younger or trained populations [16,17,18]. However, it was designed to provide a high relative stimulus for postmenopausal women with overweight/obesity. Previous studies indicate that walking at speeds of 4.5–5 km/h can represent moderate-to-vigorous intensity in sedentary individuals with obesity due to reduced functional capacity [36,37]. In our study, the HIIWT protocol elicited a high relative effort, consistent with population-specific HIIE recommendations for clinical and populations with obesity [38,39].

Postmenopausal women with overweight or obesity are particularly vulnerable to adverse changes in body composition, such as increased fat mass and reduced lean mass, as well as declines in hematological health, cardiovascular function, and overall physical capacity. Hormonal alterations and natural aging process increase the risk of metabolic and functional impairments [40,41,42]. Walking-based programs are often recommended due to their ease, accessibility, and low risk of injury also in this population [43], but optimal intensity of walking training remains unclear.

Both exercise programs resulted in significant reductions in body weight and fat mass, which is particularly significant considering that even modest weight loss has been associated with significant health benefits in adults with obesity, including increased insulin sensitivity [44] and improved lipid profiles [45], endothelial function [46], and overall quality of life [47]. Body mass loss decrement was significantly greater in the HIIWT group. This is consistent with recent evidence showing that high-intensity interval training produces significantly greater reductions in total body fat percentage compared to moderate-intensity training [48,49,50,51]. The greater fat loss observed in the HIIWT group can be attributed to greater total energy expenditure and greater post-exercise oxygen consumption, which is significantly greater after interval walking compared to continuous walking also at the same total oxygen consumption [52]. In other words, repeated high-intensity sessions likely stimulated metabolic processes (e.g., fat oxidation) more, resulting in greater adiposity losses. Intensive intermittent training is more effective than moderate-intensity training in enhancing the secretion of catecholamines, such as epinephrine [53], norepinephrine [54], and growth hormone [55], which in turn stimulate lipolysis [56,57] and promote greater fat oxidation [58], ultimately contributing to significant weight reduction. Importantly, lean mass was maintained in both groups, suggesting that the interventions promoted fat loss without loss of muscle.

Monitoring hematological parameters in postmenopausal women with obesity is of clinical relevance, as this population is at increased risk of cardiovascular dysfunction, reduced aerobic capacity, and impaired oxygen transport [59,60]. The estrogen levels decline in menopause can negatively affect blood rheology, vascular function, and fluid balance [61], potentially exacerbating the impact of obesity-related inflammation and hemodynamic stress [41]. In this context, evaluating changes in erythrocyte count, hemoglobin concentration, hematocrit, and plasma volume in response to physical training provides valuable insight into cardiovascular and metabolic adaptations, guiding the optimization of exercise interventions for improving health outcomes in this vulnerable population [1,62].

Most of the existing literature focuses on athletes undergoing intense training sessions [63]. Consistent with our results, previous research has reported reductions in erythrocytes, hemoglobin, and hematocrit, along with increased PVV, following periods of high-intensity training or competitive phases in athletes and physically active women [63,64]. These changes are generally attributed to plasma volume expansion rather than a true loss of red cell mass. This type of hematological adaptation has also been described in the context of sports pseudo-anemia, a well-known phenomenon in endurance athletes [65]. Although our participants are not athletes, the physiological mechanisms underlying this adaptation may be similar. The greater effect observed in the HIIWT group suggests that higher training intensity may induce more pronounced fluid regulatory responses, possibly through enhanced activation of hormonal mechanisms such as the renin–angiotensin–aldosterone system and the antidiuretic hormone and increased plasma protein synthesis especially albumin [5,62,66].

Regarding the responses of MCV, MCH, and MCHC, our results showed no significant changes following the training period. These findings are consistent with those reported by Saidi et al. [63], who also observed no significant alterations in these indices after a period of match congestion in professional soccer players. Although reductions were observed in erythrocyte count, hemoglobin concentration, and hematocrit levels, the stability of MCV, MCH, and MCHC suggests that erythrocyte morphology and functionality remained intact. This supports the idea that the observed hematological decreases are primarily due to plasma volume expansion (hemodilution), rather than to red blood cell loss or dysfunction [4,5,67]. This reflects a normal physiological response to aerobic training [1,4,68]. Future studies should explore whether longer or more intense training protocols induce changes in these indices, indicating deeper erythropoietic adaptations.

The present study demonstrated a significant reduction in CK and LDH levels following the HIIWT program in postmenopausal women with overweight/obesity. Conversely, no significant modifications were observed after the MIIWT. Although evidence on the effects of exercise training on muscle damage biomarkers in postmenopausal women with obesity remains scarce, our findings are in line with previous studies conducted in healthy adults and trained populations [28,69,70]. Specifically, these studies reported significant reductions in circulating levels of CK and LDH following six to eight weeks of HIIT in young healthy adults and sprinters. The observed reductions in CK and LDH are particularly relevant for postmenopausal women with obesity, as preserving muscle integrity and minimizing exercise-induced muscle damage are crucial for maintaining physical function and reducing the risk of frailty-related complications in this vulnerable population [71,72,73]. Sarcopenia—a progressive and generalized loss of skeletal muscle mass and strength—is a major contributor to frailty, impaired mobility, falls, and loss of independence in older adults, especially women [74]. Because postmenopausal women are already predisposed to accelerated muscle deterioration due to hormonal decline and sedentary behaviors, interventions that support muscle preservation without exacerbating tissue damage are of particular clinical importance [75,76].

The reduction in muscle damage markers observed following HIIWT suggests that this form of training, despite its intensity, may promote beneficial adaptations while avoiding harmful levels of mechanical stress, thereby supporting functional autonomy and improving long-term health outcomes in this vulnerable population [71].

The reduction in CK and LDH levels observed after HIIWT may reflect improved muscle membrane stability [77,78,79]. Although not directly assessed in this study, HIIT has been shown to enhance sarcolemma integrity and cytoskeletal proteins (e.g., dystrophin, desmin), limiting enzyme leakage [80]. Moreover, HIIT can strengthen antioxidant defenses, reducing oxidative stress and protecting muscle tissue from exercise-induced damage [81]. The absence of significant changes following MIIWT suggests that moderate-intensity exercise may not provide a sufficient physiological stimulus to elicit measurable reductions in muscle damage markers [82,83]. This highlights the critical role of exercise intensity in enhancing muscle resilience and maximizing the benefits of training interventions—particularly in populations vulnerable to muscle degradation, such as overweight or postmenopausal women [84,85,86].

Assessing aerobic capacity in postmenopausal women with overweight or obesity is of critical clinical importance, as it serves as a key indicator of cardiovascular and functional health [87]. This population is at heightened risk for reduced cardiorespiratory fitness due to age-related declines in estrogen levels, increased fat mass, and sedentary lifestyles, all of which contribute to diminished oxygen transport and utilization [88,89]. Lower aerobic capacity has been independently associated with greater all-cause and cardiovascular mortality, physical frailty, and reduced quality of life in older adults [90,91]. Women in the postmenopausal phase often exhibit declines in endothelial function and mitochondrial efficiency, which further compromise aerobic performance [92]. Therefore, evaluating and improving aerobic capacity in this vulnerable group is essential not only for preventing cardiometabolic diseases but also for maintaining independence and functional autonomy.

The HIIWT program led to a significant improvement in aerobic capacity, as evidenced by enhanced performance on the 6MWT. In contrast, the MIIWT program did not yield any statistically significant changes in this parameter. These results align with those of Cano-Montoya et al. [93], who reported that eight weeks of high-intensity interval training (HIIT) significantly improved 6MWT performance in older women with obesity and impaired cardiometabolic profiles. The consistency between findings suggests that higher-intensity training modalities may be more effective in eliciting functional cardiovascular adaptations in postmenopausal women with obesity. The observed improvement in aerobic capacity following the HIIWT intervention may be partially explained by two key physiological mechanisms: plasma volume expansion and reduced muscle damage. Jabbour et al. [8] demonstrated that HIIT effectively increases plasma volume, which can enhance oxygen transport and endurance capacity in postmenopausal women with obesity. At the same time, muscle damage has been identified as a limiting factor for physical performance [94]. For instance, Main et al. [95] reported that elevated markers of muscle damage were associated with reduced muscular function after prolonged endurance training. Therefore, the reductions in CK and LDH observed in our study may have contributed to improved aerobic performance by preserving muscle integrity and facilitating recovery, particularly in a population at risk of exercise-induced damage.

The absence of significant aerobic capacity gains in the MIIWT group suggests that moderate-intensity interval walking may not provide a sufficient cardiorespiratory stimulus to elicit measurable improvements in VO_2_peak or functional capacity over a 10-week period in obese postmenopausal women. Additionally, a ceiling effect may have limited detectable gains, as some participants exhibited relatively preserved baseline 6MWT values. However, some studies have reported improvements in aerobic outcomes following moderate intensity walking interventions in similar populations under certain conditions [23,96,97]. These mixed findings highlight the importance of prescribing exercise intensity relative to participants’ baseline capacity and suggest that future research should explore whether longer intervention durations, higher total training volumes, or slightly increased moderate intensities are required to achieve meaningful aerobic improvements in this population.

The present findings provide important implications for exercise prescription in postmenopausal women with overweight or obesity. Walking-based exercise is widely recommended in clinical and community settings due to its safety, accessibility, and low cost. Our results suggest that incorporating higher-intensity intervals within walking programs may provide greater physiological benefits than moderate-intensity interval walking performed with similar frequency and duration.

In particular, the HIIWT protocol resulted in greater improvements in body composition, plasma volume expansion, hematological parameters, muscle damage markers, and aerobic capacity. These adaptations are clinically relevant, as improved oxygen transport, cardiovascular efficiency, and muscle integrity may help counteract the functional decline commonly observed in postmenopausal women with obesity.

Importantly, exercise intensity in this study was individualized using the 6 min walk test, providing a simple and practical method for prescribing training intensity in settings where advanced physiological monitoring is not available. Therefore, interval walking based on individual functional capacity may represent an effective and feasible strategy to improve cardiometabolic health and physical function in this population.

Several limitations of this study must be acknowledged. Although the minimum sample size was statistically calculated, the cohort limits the generalizability of the findings to the broader population of postmenopausal women with overweight or obesity. Another notable limitation is the lack of assessment of indicators of iron status, such as serum ferritin and serum sideremia. Given their central role in erythropoiesis and hemoglobin regulation, the omission of these markers limits our interpretation of hematologic adaptations, particularly in a population in which iron metabolism may be altered by age and hormonal status. Finally, although the study assessed multiple physiological responses, it did not investigate the endocrine and renal mechanisms underlying plasma volume expansion, such as activation of the renin angiotensin aldosterone system or fluctuations in antidiuretic hormone levels, thus limiting the mechanistic understanding of adaptations to fluid regulation. It should be noted that, given the population of postmenopausal women with obesity, the intensities achieved in the HIIWT protocol may not correspond to classical HIIT thresholds reported in younger or athletic adults [16,17,18].

Therefore, while HIIWT elicited greater short-term improvements in several outcomes compared to MIIWT, these findings should be interpreted as preliminary and indicative of short-term training adaptations rather than definitive evidence of superior efficacy.

## 5. Conclusions

HIIWT was associated with greater improvements in selected physiological outcomes compared to MIIWT in postmenopausal women with overweight/obesity. Specifically, HIIWT led to greater improvements in body composition, aerobic fitness, plasma volume expansion, and reductions in hematological and muscle damage markers. The reductions in hematological markers reflect exercise-induced plasma volume expansion (hemodilution) and are physiologically beneficial, supporting enhanced oxygen transport and muscular integrity. Overall, these adaptations contributed to better functional capacity. Therefore, HIIWT appears to be an effective strategy to improve cardiometabolic health and physical function in this high-risk population, achieving greater benefits than MIIWT within the same session duration. Future studies with longer follow-up and more precise intensity monitoring—e.g., VO_2_ calibration, HRR, or continuous wearable tracking—along with assessment of hormonal mediators of plasma volume regulation, are warranted to confirm these findings and further elucidate the underlying physiological mechanisms.

## Figures and Tables

**Table 1:** Energy intake and macronutrient composition in HIIWT, MIIWT, and CON groups.

Group	Energy Intake (kcal/Day)	Protein (g/Day)	Fat (g/Day)	Carbohydrates (g/Day)
CON	1990 ± 208	90 ± 8	66 ± 7	250 ± 15
MIIWT	2020 ± 228	91 ± 9	70 ± 8	258 ± 18
HIIWT	1995 ± 212	90 ± 7	67 ± 6	259 ± 16

Note: No specific dietary restrictions were applied, and intake was maintained throughout the study. Values are means ± SD based on 3-day food diaries collected at multiple time points. No significant differences were observed between groups (*p* > 0.05, ANOVA).

**Table 2:** Pre- and post-intervention anthropometric and biochemical parameters in HIIWT, MIIWT, and CON group.

	CON Group (*n* = 10)		MIIWT Group (*n* = 11)		HIIWT Group (*n* = 11)					
	Pre	Post	Pre	Post	Pre	Post				
Age (years)	49.20 ± 3.46		50.36 ± 4.39		50.18 ± 5.02		Pre	Interaction (time × group)		
Height (m)	1.58 ± 0.07		1.59 ± 0.06		1.61 ± 0.04		*p*	F	*p*	η_p_^2^
Body mass (kg)	83.7 ± 9.58	84.0 ± 10.1	84.2 ± 7.31	83.3 ± 7.28 *	83.4 ± 7.42	79.4 ± 6.53 ***	0.977	16.469	<0.001	0.532
Body mass index (kg/m^2^)	33.4 ± 3.63	33.5 ± 3.82	33.4 ± 3.29	33.1 ± 3.29 *	32.3 ± 3.21	30.8 ± 3.11 ***	0.718	17.535	<0.001	0.547
Body fat (%)	44.4 ± 4.32	44.6 ± 4.60	43.6 ± 5.26	43.2 ± 5.51 *	43.1 ± 6.72	41.5 ± 6.32 ***	0.868	13.711	<0.001	0.486
Lean mass (kg)	46.5 ± 4.58	46.6 ± 4.60	47.2 ± 4.10	47.3 ± 4.12	46.9 ± 4.33	46.9 ± 4.35	0.922	0.85	0.44	0.002
Waist circumference (cm)	124 ± 12.9	125 ± 12.8	124 ± 8.30	122 ± 7.33 *	122 ± 7.09	117 ± 7.72 **	0.871	6.157	0.006	0.298
Erythrocytes (10^3^/μ L)	4.80 ± 0.25	4,88 ± 0.29	4.85 ± 0.30	4.74 ± 0.23 *	4.88 ± 0.19	4.69 ± 0.15 *	0.745	6.516	0.005	0.310
Hemoglobin (g/d L)	12.8 ± 0.70	13.0 ± 0.72	12.9 ± 0.72	12.7 ± 0.61	13.2 ± 0.60	12.7 ± 0.50 ***	0.428	3.345	0.049	0.187
Hematocrit (%)	41.2 ± 1.93	41.5 ± 1.26	41.1 ± 1.56	40.9 ± 1.88	42.6 ± 4.71	40.3 ± 3.23 **	0.505	3.658	0.038	0.201
MCV (fl)	82.9 ± 3.05	83.1 ± 2.76	83.9 ± 5.79	83.8 ± 5.08	82.8 ± 6.85	82.7 ± 5.69	0.887	0.005	0.995	0.000
MCHC (pg)	28.6 ± 1.45	28.7 ± 1.26	28.6 ± 1.59	28.5 ± 1.14	28.2 ± 2.41	28.3 ± 2.15	0.865	0.022	0.979	0.001
MHC (g/d)	32.1 ± 1.92	32.6 ± 1.98	32.4 ± 1.44	32.1 ± 1.86	32.9 ± 1.45	32.3 ± 2.41	0.528	0.705	0.502	0.046
6MWT distance	452 ± 24.6	451 ± 27.2	456 ± 18.7	458 ± 15.8	457 ± 22.6	464 ± 19.1 **	0.848	3.475	0.044	0.193

Data are expressed as mean ± SD. MCV, Mean Corpuscular Volume; MCHC, Mean Corpuscular Hemoglobin Content; MHC, Mean Hemoglobin Concentration; PVV, Plasma Volume Variation; 6MWT, 6-min walk test. * *p*  <  0.05; ** *p*  <  0.01; *** *p*  <  0.001 based on post hoc tests after a significant time × group interaction (two-way repeated measures ANOVA, ηp^2^ reported). No significant baseline differences were observed between groups (one-way ANOVA, *p* > 0.05).

## Data Availability

The data supporting the findings of this study are not publicly available due to privacy and ethical restrictions. However, they can be accessed upon reasonable request from the corresponding author.

## Abbreviations

The following abbreviations are used in this manuscript:

HIIWT	High-intensity interval walking training
MIIWT	Moderate-intensity interval walking training
6MWT	6 min walk test
LDH	Lactate dehydrogenase
CK	Creatine kinase
